# Genetic Analysis of 252 Index Cases with Inherited Retinal Diseases Using a Panel of 351 Retinal Genes

**DOI:** 10.3390/genes15070926

**Published:** 2024-07-16

**Authors:** Maria Abu Elasal, Samira Mousa, Manar Salameh, Anat Blumenfeld, Samer Khateb, Eyal Banin, Dror Sharon

**Affiliations:** Division of Ophthalmology, Hadassah Medical Center, Faculty of Medicine, The Hebrew University of Jerusalem, Jerusalem 91120, Israel; maria.abuelasal@mail.huji.ac.il (M.A.E.); samira.mousa@mail.huji.ac.il (S.M.); manar.salameh@mail.huji.ac.il (M.S.); anatbl@savion.huji.ac.il (A.B.); samerkhateb@gmail.com (S.K.);

**Keywords:** consanguinity, gene panel, inherited retinal diseases, retinal dystrophy, targeted next-generation sequencing

## Abstract

Inherited retinal diseases (IRDs) are extremely heterogeneous with at least 350 causative genes, complicating the process of genetic diagnosis. We analyzed samples of 252 index cases with IRDs using the Blueprint Genetics panel for “Retinal Dystrophy” that includes 351 genes. The cause of disease could be identified in 55% of cases. A clear difference was obtained between newly recruited cases (74% solved) and cases that were previously analyzed by panels or whole exome sequencing (26% solved). As for the mode of inheritance, 75% of solved cases were autosomal recessive (AR), 10% were X-linked, 8% were autosomal dominant, and 7% were mitochondrial. Interestingly, in 12% of solved cases, structural variants (SVs) were identified as the cause of disease. The most commonly identified genes were *ABCA4*, *EYS* and *USH2A*, and the most common mutations were *MAK*-c.1297_1298ins353 and *FAM161A*-c.1355_1356del. In line with our previous IRD carrier analysis, we identified heterozygous AR mutations that were not the cause of disease in 36% of cases. The studied IRD panel was found to be efficient in gene identification. Some variants were misinterpreted by the pipeline, and therefore, multiple analysis tools are recommended to obtain a more accurate annotation of potential disease-causing variants.

## 1. Introduction

Inherited retinal diseases (IRDs) encompass a broad spectrum of retinal phenotypes characterized by extensive clinical variability and profound genetic heterogeneity [1,2,3]. Among IRDs, retinitis pigmentosa (RP) stands out as one of the most prevalent and heterogeneous conditions in humans, caused by disease-causing mutations in over 60 genes contributing to its etiology. The inheritance patterns of RP include autosomal recessive (AR) in 50–60%, autosomal dominant (AD) in 30–40% of cases, and X-linked (XL) in 5–15%, along with rare digenic and mitochondrial modes [2,4,5,6,7,8,9].

RP is a progressive inherited retinal degenerative disease primarily affecting rod photoreceptors cells, followed by cone photoreceptors. Most patients initially experience night blindness, which progresses to a gradual loss of visual acuity and narrowing of the visual field [1,2,4,5,6,7]. The condition can be classified into syndromic RP, which involves multiple organ systems leading to significant dysfunction, and non-syndromic RP, accounting for 70–80% of cases, in which only the retina is affected [4].

Currently, there is no effective treatment for the vast majority of IRDs, although this field has led to breakthroughs in developing therapeutic modalities for inherited diseases. Over the last two decades, gene augmentation therapy has been proved efficient in numerous animal models for IRDs [2] and the first-in-man gene augmentation therapy was approved by the Food and Drug Administration (FDA) in 2017 for biallelic *RPE65* pathogenic variants [10]. The success of this treatment led to the development of other therapeutic modalities for IRDs, such as CRISPR [11] and antisense oligonucleotide treatment [12].

The advent of substantial sequencing breakthroughs over the past few decades as well as improved bioinformatics and functional analyses has heralded a remarkable advancement in mutation detection methods as well as mutation characterization [13]. These include next-generation sequencing (NGS), whole exome sequencing (WES), whole genome sequencing (WGS), functional genomic techniques (such as CRISPR-Cas9 gene editing, RNA sequencing, and functional assays), bioinformatics analysis, single-cell genomics, epigenomic profiling techniques (including ChIP-seq and DNA methylation profiling), and multi-omics integration. Each of these modern methodologies offers unique advantages in elucidating the genetic underpinnings of various diseases.

From providing a comprehensive view of disease mechanisms to facilitating the identification of novel mutations and understanding complex diseases with heterogeneous genetic backgrounds, these techniques are often employed synergistically to enhance diagnostics, tailor personalized treatments, and refine disease management strategies.

However, the selection of an appropriate method is fraught with challenges due to several inherent limitations. These include cost considerations, the complexity of data analysis, the potential for false positives and false negatives, limitations in functional validation, incomplete genomic coverage, ethnic diversity, ethical and privacy concerns surrounding genomic data, and the intricacies of clinical utility and interpretation.

In light of these challenges, we endeavor to navigate the landscape of available methodologies, striving to identify the most suitable approach that aligns with our goal of providing comprehensive genetic insights to many patients. Our aim is to unveil all pertinent mutations contributing to the heterogeneity of IRDs.

Here, we used a single gene panel to screen the DNA of 252 unsolved cases with IRDs. The analysis revealed the identification of the cause of disease in 55% of cases, with variability in detection rates between sub-groups.

## 2. Materials and Methods

### 2.1. Patient Recruitment and Clinical Analyses

Our cohort of patients with IRD at the Hadassah Medical Center includes over 2300 Israeli and Palestinian families that were recruited in the last 25 years. For the current study, participating patients were recruited between 2006 and 2022, some of whom were genetically evaluated prior to the current study using various techniques as detailed below and in Table S1. Blood and/or saliva samples were collected from 252 patients with IRDs, representing diverse ethnic backgrounds, genders, ages, and retinal conditions (Table S1). This sub-cohort included newly recruited patients that did not undergo previous genetic analysis (*n* = 98) and those who remained unsolved following previous screening tests (*n* = 154). From the newly recruited group, saliva samples or DNA that was extracted from blood (see below) were shipped to Blueprint Genetics, and from the previously screened group, DNA that was extracted from blood was shipped.

The sample collection process adhered to ethical standards, receiving explicit approval from the Hadassah Hospital Institutional Review Board. Informed consent, obtained in writing, ensured compliance with all relevant ethical regulations and safeguarded the rights and privacy of participants.

The following parameters were calculated in this study: diagnostic success rates (% of solved cases), modes of inheritance (% of each inheritance pattern following gene identification), the presence of structural variants (SVs; % of SV alleles out of the total number of mutated alleles), and the distribution of affected genes (% of cases solved by a specific gene out of all solved cases).

A few cases, representing either rare conditions or elusive sequence variants, were selected for detailed clinical description. Ophthalmic evaluation included a full ophthalmological examination, Goldmann perimetry (using Humphrey or Octopus systems), full-field electroretinography (FFERG) according to the ISCEV standard [14] (using LKC or Diagnosys systems), color vision testing using the Ishihara 38-panel and Farnsworth–Munsell D-15 tests, and color, autofluorescence and OCT imaging using Optos, (Marlborough, MA, USA), TOPCON (Capelle aan den Ijssel, The Netherlands), Eidon (Vantaa, Finland), and Heidelberg Spectralis systems (Franklin, MA, USA).

### 2.2. DNA Extraction

Genomic DNA was extracted from blood samples using the Promega kit and a Promega Maxwell DNA extraction device (Promega, Madison, WI, USA). For DNA extraction from saliva, buccal swab samples were collected from patients using the OracolectDNA OCR-100 Saliva Kit (DNA Genotek, Ottawa, ON, Canada). DNA concentration was examined using a Nanodrop microvolume spectrophotometer (ThermoFisher; Waltham, MA, USA) and 1% agarose gel staining.

### 2.3. Next-Generation Sequencing (NGS)

The retinal dystrophy panel (Blueprint Genetics; Espoo, Finland; test code OP0801: https://blueprintgenetics.com/tests/panels/ophthalmology/retinal-dystrophy-panel/; accessed on 30 June 2024), including 351 IRD genes (Table S2), was used to analyze the DNA of 252 IRD cases. Clinical-grade NGS on the Illumina NovaSeq system was employed using the NovaSeq 6000 kit, allowing comprehensive coverage across various genomic IRD genes covering 99.86% of the target region, with a minimal depth of >20×. Raw sequencing data were transformed into FASTQ format using Illumina’s software bcl2fastq v.2.20 and mapped to the human reference genome (GRCh37/hg19). Burrows-Wheeler Aligner (BWA-MEM v1.1.5) software was used for read alignment. Duplicate read marking, local realignment around indels, base quality score recalibration and variant calling were performed using the GATK algorithm. Variant data were annotated using a collection of tools (VcfAnno and VEP) with a variety of public variant databases including gnomAD, ClinVar and HGMD. The analysis included the detection of single-nucleotide variations (SNVs), SVs (defined as a single exon or larger deletions or duplications), insertions, deletions, and indels. Median sequencing depth was 80× at the nucleotide level, ensuring unparalleled sensitivity and accuracy in variant detection. Each interpretation obtained by the Blueprint pipeline was re-evaluated using population-specific information using prior available information on allele frequency in the Israeli and Palestinian IRD cohorts.

The detection performance of the panel yielded high results. The following sensitivity values were obtained: for nuclear DNA: 99.89%, for SNVs detection—99.2% for indels of 1–50 bps, 100% for a single exon deletion, and 98.7%, for 5 exons SVs, with high specificity value of >99.9% for most variant types; for mtDNA: 100.0%, 94.7%, and 87.3% for SVs and indels (10–100% heteroplasmy level, 5–10% heteroplasmy level, and <5% heteroplasmy level; respectively), and 100.0% for gross deletions, with specificity of >99.9% for all.

## 3. Results

### 3.1. Patient Demographics

**Figure 1:**
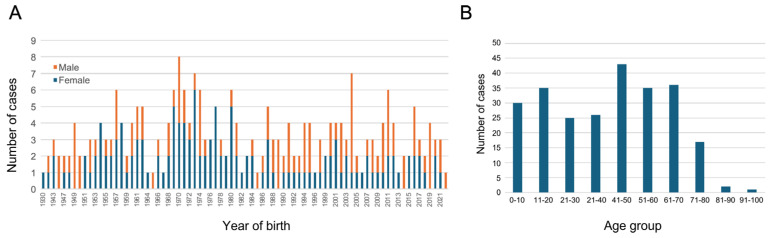
Demographic representation of the studied sub-cohort of 252 IRD cases. (**A**) A bar graph representing the distribution of the year of birth of males and females who participated in the study. (**B**) A histogram representing the number of participants in each age group.

A total of 252 index cases, selected from a cohort of over 2300 families, were enrolled in this study. Of these, 230 DNA samples were extracted from blood, with 98 having undergone testing with a negative output. Additionally, 22 fresh saliva samples from newly recruited patients were shipped for analysis. Our cohort exhibited ethnic diversity, with 66% Jews (including 26% Ashkenazi Jews and 12% North African Jews), 31% Arab Muslims (including 3% Bedouins), and other ethnicities with less than 1% each. Gender and age distribution (Figure 1) show uniform recruitment from an early age through the age of 80 years, including both males and females at relatively similar ratios, with a higher percentage of females (54% vs. 46% for males).

### 3.2. Efficiency of the Gene Panel

**Figure 2:**
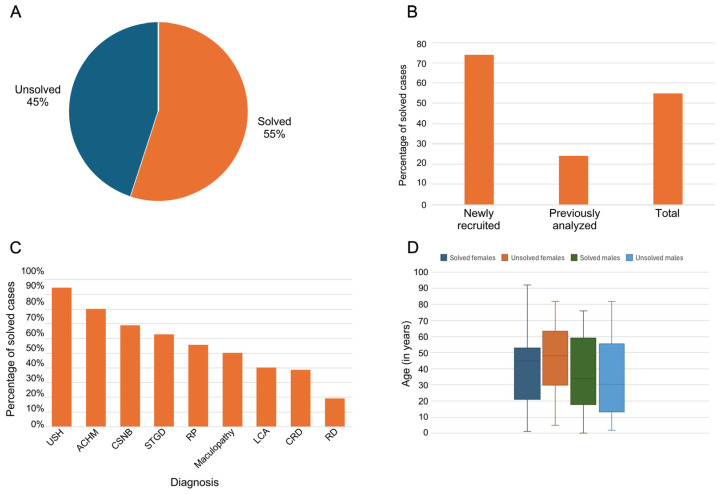
Insights into the effectiveness of the gene panel in different diagnosis histories. (**A**) A pie chart representing the distribution of solved and unsolved cases among a total of 252 samples analyzed using the Blueprint retinal dystrophy gene panel. (**B**) A bar chart that compares the success rates in comparison to two distinct groups: newly recruited samples and previously analyzed samples with negative results (please see Table S1 for more details). (**C**) A bar graph representing the percentage of solved cases for different IRD phenotypes. Only phenotypes with n > 4 are presented. (**D**) A box and whisker plot representing the age distribution among solved and unsolved males and females. ACHM—achromatopsia, CRD—cone-rod degeneration, CSNB—congenital stationary night blindness, LCA—Leber congenital amaurosis, RD—retinal degeneration, RP—retinitis pigmentosa, STGD—Stargardt disease, USH—Usher syndrome.

The genetic cause of disease (pathogenic or likely pathogenic mutation/s) was identified in 138 out of the 252 analyzed samples, with a yield rate of 55% (Figure 2A and Table S1). Consequently, 114 cases remained unsolved (Figure 2A and Table S1). Further analysis revealed varying rates of gene identification between two distinct groups: newly recruited samples exhibited a 74% success rate, while previously analyzed samples with negative results (the analysis included genotyping of founder mutations, IRD gene panels, and WES) showed a 24% success rate (Figure 2B). Out of the 138 solved cases, 50 (36%) carry a heterozygous AR mutation in another IRD gene, and out of the 114 unsolved cases, 52 (46%) are carriers.

In 11 cases (Table S3), variant interpretation by the Blueprint pipeline was revised, mainly due to data that were not available in the literature. This includes five cases in which the initial interpretation concluded that these cases were solved but additional information contradicted it and six cases in which the revised analysis concluded that the variant was pathogenic. For example, case MOL1839-1 carried three heterozygous variants in the AR gene *ABCA4*, two were interpreted as likely pathogenic and one as a variant of unknown significance (VUS). However, the patient phenotype (congenital stationary night blindness—CSNB) did not fit this causative gene and the variants did not cosegregate with the phenotype in the family, leading to the conclusion that the ABCA4 was not the cause of disease in this case. On the other hand, case MOL1970-1 of Ashkenazi Jewish origin was diagnosed with RP and a VUS in the *CFAP410* gene was identified. In gnomAD, this variant was found mainly in Ashkenazi Jewish cases, none of them were homozygous, with an MAF of 0.51%. However, in our cohort, patients from three different families who shared the same phenotype were homozygous for this variant, and therefore, we concluded that it was likely to be a photogenic variant.

### 3.3. Charecarization of Solved Cases by Inheritance Pattern, Gene, and Phenotype

**Figure 3:**
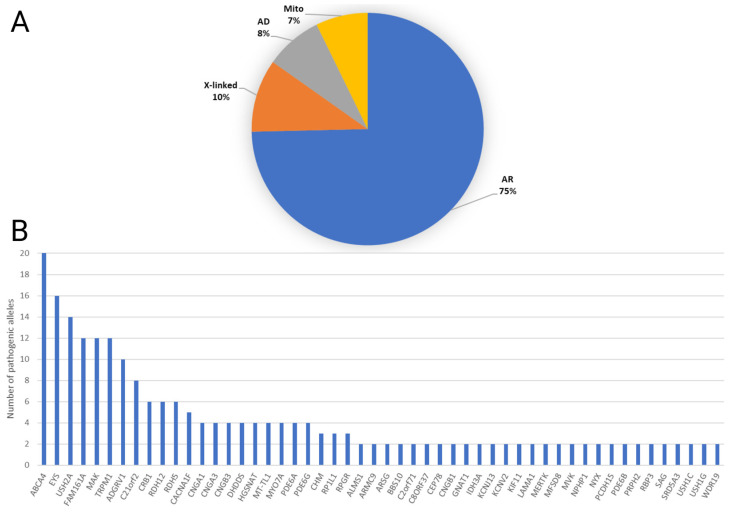
Overview of genetic mechanisms in inherited retinal diseases. (**A**) Pie chart illustrating the distribution of solved cases based on the mode of inheritance, including autosomal recessive (AR), X-linked, autosomal dominant (AD), and mitochondrial genes. Each segment represents the percentage of cases solved under each mode of inheritance. (**B**) Bar chart presenting the frequency of the most implicated genes in the cohort of 138 solved cases. The *X*-axis displays the frequently identified genes, while the *Y*-axis represents the number of alleles in families for which the gene was identified as the cause of their phenotype.

The majority of solved cases were attributed to an autosomal recessive (AR) inheritance pattern (75%), followed by X-linked (10%), autosomal dominant (AD) (8%), and mitochondrial (7%) (Figure 3A). The most frequently implicated genes were *ABCA4* accounting for 8%, *EYS* with 7%, and *USH2A* with 6% (Figure 3B). Five phenotypes (with at least five cases each) showed a relatively high proportion of solved cases (USH, ACHM, CSNB, STGD, and RP; Figure 2C), with more than 50% solved. No difference was evident between the mean age of solved and unsolved cases (Figure 2D).

### 3.4. Frequency of the Most Common Mutations

**Figure 4:**
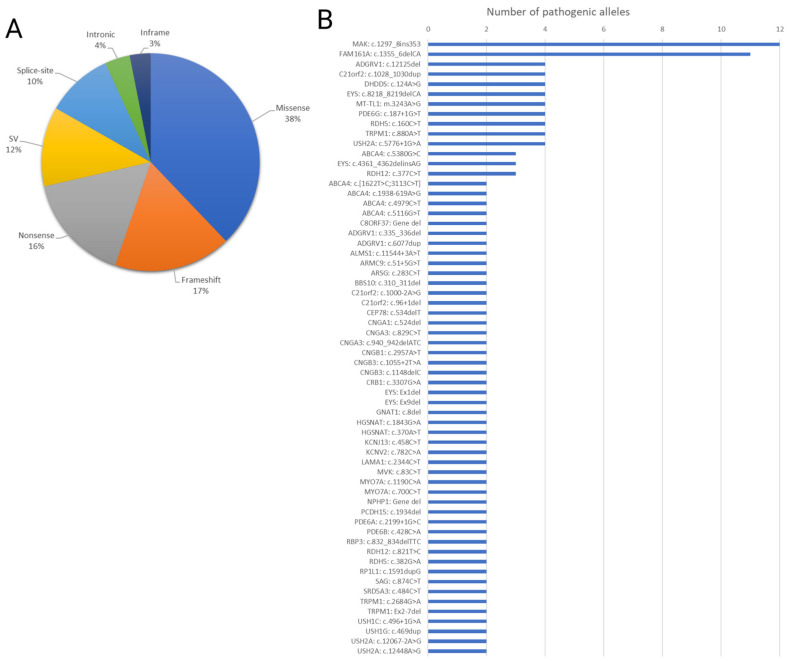
Spectrum of genetic alterations contributing to inherited retinal diseases. (**A**) Pie chart visually representing the distribution of various mutation types identified in the solved cases, including missense, frameshift, nonsense, structural variants (SVs), splice site, intronic, and in-frame mutations. Each segment depicts the percentage of cases attributed to a specific mutation type. (**B**) Bar chart depicting the number of alleles for each of the most recurrent mutations detected among the indexed cases. The *X*-axis displays the identified disease-causing variants and the *Y*-axis represents the number of alleles in families for which the mutation was identified as the cause of their phenotype.

Missense mutations constituted the most common variant type (38%), followed by frameshift (17%), nonsense (16%), structural variants (SVs) (12%), splice-site (10%), intronic (4%), and in-frame mutations (3%) (Figure 4A). Notably, the most recurrent mutations identified were c.1297_1298ins353 in *MAK* and c.1355_1356del in *FAM161A* (Figure 4B).

### 3.5. Case Details of Rare Phenotypes and Elusive Mutations

We selected a few cases aiming to present the variability of the studied cohort, rare cases with regard to both phenotype and genotype, and elusive mutations that might be missed without the proper knowledge.

#### 3.5.1. KIF11 and Non-Syndromic Chorioretinopathy

The *KIF11* gene encodes a homo-tetrameric motor protein crucial for spindle polarity during mitosis. Variants in this ubiquitously expressed protein are typically de novo and associated with various developmental syndromes, including impacts on retinal vasculature development. Considered rare, only one family in Israel is documented with a *KIF11* mutation [15]. Surprisingly, among the 142 cases solved in our study, *KIF11* emerged as the causal gene in two unrelated Ashkenazi Jewish patients.

Case MOL1984-1 was diagnosed at the age of 10 years with reduced visual acuity, mild myopia (−3 D), and photophobia. His best corrected visual acuity (BCVA) at the age of 16 was 0.6/0.5 in the right and left eye, respectively, and OCT showed a retinoschisis pattern. He has no family history of visual deficiencies. An ERG test at the age of 18 years revealed sub-normal responses, including cone flicker, mixed cone-rod b-wave, and rod responses. Color vision was within the normal range. An ophthalmic evaluation at the age of 19 years revealed atrophic chorioretinal patches next to the optic disk and the inferior arcade. Following a clinical evaluation at the age of 19, no other clinical features were identified in this case, as reported in a few other *KIF11* cases [16]. The patient was therefore diagnosed with non-syndromic chorioretinopathy. Interestingly, gene panel analysis revealed a heterozygous frameshift mutation in *KIF11* (Table S1).

Case MOL2101-1 was diagnosed with a congenital retinal disease, including chorioretinal scars and retinoschisis. Visual acuity deteriorated and at the age of 11 reached 0.4 in the right eye and hand motion in the left eye with mild myopia (−3.5 diopters). Multiple electroretinography (ERG) examinations revealed non-progressive sub-normal ERG responses, including cone flicker, mixed cone-rod a and b-wave and rod responses. Following multiple clinical evaluations between ages 1 and 19 years, no other clinical features were identified in this case. The patient was therefore diagnosed with non-syndromic chorioretinopathy. Interestingly, gene panel analysis revealed a heterozygous de novo nonsense mutation in *KIF11* (Table S1).

#### 3.5.2. An Elusive Coding Mutation

Further illustrating the complexities of genetic diagnostics, an intriguing and elusive nonsense pathogenic mutation in the *EYS* gene was identified.

The index case, MOL2142-1, was diagnosed with ARRP due to extinguished ERG responses at the age of 55 years, a fundus appearance that included typical signs of RP (bone-spicule pigmentation and attenuated blood vessels), and a reduced BCVA of 0.25. An analysis of SVs in IRD genes, as part of the panel analysis, revealed a heterozygous deletion of a single *EYS* exon (#34), as well as two nucleotide changes (one synonymous and the other nonsynonymous), affecting neighboring nucleotides (c.4361C>A, p.S1454Y and c.4362C>G, p.S1454=). Interestingly, this combination of variants was identified in multiple cases with an RP of Arab Muslim origin and confirmed to be in cis. The combination of the two variants results in a nonsense mutation (c.4361_4362delinsAG, p.S1454*; Table S1). Such a complex mutation can be overlooked in many NGS pipelines, highlighting the challenges of variant interpretation and the importance of comprehensive genetic panels.

#### 3.5.3. Novel Genetic Discoveries in Israeli Patients: Unveiling Rare Variants and First Cases

Joubert syndrome, a recessive and genetically heterogeneous neurodevelopmental ciliopathy, is characterized by a distinctive brain malformation, often presenting with a complex phenotype that may include retinal dystrophy. In our study, we identified two cases, MOL2246-1 and MOL22151-1, marking the first documented instances in Israel of Joubert syndrome associated with mutations in the *ARMC9* and *LAMA1* genes, respectively. Notably, these genes are rare contributors to this syndrome; in the literature, only 19 cases of *ARMC9* mutations have been reported across five different articles from Japanese [17,18], Chinese [19], Turkish [20], and Indian [21] populations, while *LAMA1* mutations have been documented in just five cases from British [22] and Japanese [23] cohorts spanning two articles. This underscores the rarity and global diversity of genetic variants contributing to Joubert syndrome.

Case MOL2246-1 was diagnosed with a congenital syndrome due to polydactyly (of both fingers and toes), nephrolithiasis, dysmorphism, hypotonia, developmental delay, patent foramen ovale in the heart, ocular gaze abnormality, and hypermetropia (+5.0 diopter). An ERG examination at the age of 7 months was performed under anesthesia and revealed reduced cone function in the right eye (to 60% of lower normal limit). The parents of the index case were consanguineous of Bedouin origin and no other affected individuals were reported in the family. The genetic analysis revealed a homozygous splice-site variant, c.51+5G>T (IVS2+5G>T) in the *ARMC9* (NM_025139.6) gene. This variant has already been reported in an Israeli patient with similar symptoms [16], and in addition, it has been reported as a VUS (two reports), LP (likely pathogenic) and P (one report each) in ClinVar. It is predicted to affect splicing by multiple splicing analysis tools (SpliceAI, dbscSNV Ada, and dbscSNV RF).

Case MOL2215-1 was diagnosed with molar tooth sign, dysplastic cerebellum, developmental delay, growth delay, high myopia (−17 diopters), and esotropia. His visual acuity at the age of 5 years was 6/45 both eyes and latent horizontal nystagmus was noted. ERG testing at the age of 11 years showed low rod (75% of normal) and cone (30% of normal) amplitudes. The parents of the index case are consanguineous of Arab-Muslim origin and no other affected individual were reported in the family. The genetic analysis revealed a homozygous splice-site variant, c.2344C>T (p.R782*), in the *LAMA1* (NM_005559.4) gene. The variant has already been reported in two patients with similar phenotypes [22] and is expected to create a pre-mature stop codon in exon 17 out of 62 exons.

In addition to the aforementioned cases, our study unveiled several other rare variants, each representing the first documented instances in Israel. One such case is patient MOL1437-1, who exhibits a compound heterozygosity for missense mutations in the *WDR19* gene, resulting in non-syndromic RP. Another noteworthy finding is a homozygous nonsense mutation in the SAG gene in patient MOL2008-1, leading to Oguchi disease. Lastly, patient MOL2079-1 was identified as homozygous for a nonsense mutation in the *SRD5A3* gene, which is associated with a congenital disorder of glycosylation [24]. The patient was 33 years old and was diagnosed with RP associated with learning difficulties. ERG testing at the age of 20 years showed extinguished rod and cone responses. These five cases represent novel discoveries in the Israeli population, highlighting the significance of our study in expanding the understanding of rare genetic variants and their clinical implications.

## 4. Discussion

In the current study, we used a single IRD gene panel to screen 252 IRD samples for mutations in 351 IRD-causing genes. Previous studies reported a yield of positive genetic results in 40–75% of analyzed cases and our study, with 55%, falls within this range as expected [25,26,27,28,29]. Our cohort could be divided into newly recruited cases who were not screened for any IRD-causing mutations and those who underwent genetic analysis (including genotyping founder mutations, IRD gene panels, and WES). The power of the current panel analysis can be appreciated by the much higher detection rate of 74% compared to only 26% of those previously analyzed (mainly due to mutations in genes that were not included in the previous analyses or SVs). This high rate can partly be attributed to SV analysis performed on the NGS data. Improved recent NGS analysis pipelines include the detection of both homozygous or heterozygous SVs, which assist in the identification of such mutations on 10–15% of pathogenic alleles [30,31,32]. Switching to WGS is likely to increase this rate and improve the overall detection rate of pathogenic alleles in IRDs.

The genetic analysis of newly diagnosed IRD cases depends on the phenotypic complexity and the available information on IRDs in the relevant population. The spectrum of IRD mutations in the Israeli population was studied comprehensively, mainly by a nationwide collaborative effort of the Israeli inherited retinal diseases consortium (IIRDC), leading to enriched information on the disease prevalence of various IRD phenotypes [33], the development of an NGS founder mutation gene panel [34], and the identification of a large number of mutations in each sub-population [35,36,37]. This continuous effort allows us, in some cases, to pinpoint the pathogenic mutation quickly, without the need of time-consuming and costly analyses. However, in the majority of cases, either due to the lack of known common founder mutations or challenging phenotyping, gene panels, such as the one used in the current study, can aid in the identification of the cause of disease in about 75% of cases.

Another factor that makes the process of gene identification even more challenging is the relatively high carrier frequency rate for mutations that cause IRDs. We have shown previously using worldwide datasets that, on average, 36% of healthy individuals are carriers of AR-IRD pathogenic mutations [38]. This high value is also valid for solved IRD cases who carry an AR mutation in a second IRD gene by chance. Therefore, identifying a single heterozygous AR mutation in a patient with IRD does not necessarily mean that this gene is indeed the cause of disease. In line with this previous IRD carrier analysis, we identified here heterozygous AR mutations that were not the cause of disease in 36% of solved cases.

Quick and reliable gene identification is important for a more efficient genetic counseling and disease prevention in coming generations [39,40], participation in gene/mutation-specific clinical trials [2,41], or FDA-approved IRD therapies [42]. The analysis performed in this study shows that gene panels can be highly efficient in identifying the cause of disease and, in some cases, identifying also additional IRD-causing mutations that one out of three patients with IRD carry by chance, as we previously reported [38]. Including the mitochondrial genome and performing SV analyses on the NGS data further improves the yield of gene and mutation identification, and therefore, such panels are highly recommended.

## Data Availability

Data of this study are presented within the article and its Supplementary Materials. The authors are willing to share materials, datasets, and protocols used in the acquisition of data presented in this publication with other researchers upon request (contact Dror Sharon, e-mail: dror.sharon1@mail.huji.ac.il).

## Supplementary Materials

The following supporting information can be downloaded at: https://www.mdpi.com/article/10.3390/genes15070926/s1, Table S1: Identified gene and mutations in studied cases; Table S2: Genes included in the Blueprint Genetics Retinal Dystrophy Panel (version 7, 30 October 2021) used in the current study; Table S3: Cases in which the original and final interpretations do not match.
