# Supplementary material for: Genetic Analysis of 252 Index Cases with Inherited Retinal Diseases Using a Panel of 351 Retinal Genes
Source: Genes (Basel). 2024 Jul 16;15(7):926. doi: 10.3390/genes15070926 (PMC11276581; doi:10.3390/genes15070926)
Supplement: Supplementary file 1 [file genes-15-00926-s001.zip › Table S3.pdf]

Table S3: Cases in which the original and final interpretations do not match

Case	Phenotype	BP Primary findings	Additional Data	Final decision
1839-1	CSNB	Heterozygous for 2 pathogenic ABCA4 variants: c.5882G>A (p.G1961E) and c.286A>G (p.N96D) and a VUS: c.742G>A (p.V248M)	Phenotype (CSNB) does not fit ABCA4-disease. The 2 mutations are in cis and ABCA4 does not cosegregate.	Case is not solved
1869-1	STGD	Heterozygous for 2 CDHR1 variants: c.783G>A (p.P261=) which is pathogenic and c.143C>A (p.T48N) which is a VUS.	c.783G>A (p.P261=) has conflicting classifications of pathogenicity in ClinVar: Pathogenic (2); Likely pathogenic (6); Uncertain significance (8). MAF in Europeans reached 0.6% with 4 homozygotes. It might represent a hypomorphic allele	Case is not solved
1970-1	RP	Homozygous for CFAP410 c.671_673dup (p.L224dup) which is a VUS.	High MAF (0.51%) only in Ashkenazi Jews with no homozygotes in gnomAD. The variant was identified homozygously in at least 3 families with RP.<br>we concluded LP	Case is considered solved
2058-1:	RP	Heterozygous for KCNV2 c.959G>A (p.R320H) which is a VUS	Variant is a VUS and gene does not match the phenotype	Case is not solved
2080-1	RP	homozygous for PDE6B c.428C>A (p.A143D) which is a VUS	We identified 12 patients with RP from 5 families who are homozygous for this variant. We interpret it as pathogenic.	Case is considered solved
2136-1	RP	hemizygous for CACNA1F c.1219T>C (p.W407R) which is a VUS	We identified it in 2 XL families with 4 patients affected by RP. We interpret it as pathogenic.	Case is considered solved
2139-1	RP	Heterozygous for 2 VUSs in PDE6A: c.2125G>A (p.E709K) and c.1270A>C (p.T424P).	Both variants are rare with no homozygotes in gnomAD. c.2125G>A (p.E709K) affects a functional domain with pathogenic predictions.<br>c.1270A>C (p.T424P) was found homozygously in another RP case in our cohort.	Case is considered solved
2145-1	CSNB	Homozygous for RBP3 c.832_834del (p.F278del) which is a VUS.	c.832_834del (p.F278del) is a rare variant, adfecting a protein domain and identified in multiple CSNB patients in a homozygous state.	Case is considered solved
2143-1	CHM	Heterozygous for RS1 c.460C>G (p.Q154E) which is likely pathogenic.	There is no evidence in the literature that this variant is pathogenic. We identified in a hemizygous state in a male with	Case is not solved

			maculopathy due to a PRPH2 mutation. We therefore do not consider it pathogenic.	
2191-1	RD	Heterozygous for 2 ABCA4 VUS variants: c.3050+362_3050+370del and c.452T>C (p.I151T).	c.3050+362_3050+370del is a deep intronic likely benign variant by all prediction tools.	Case is not solved
2220-1	RP	Homozygous for USH2A c.12448A>G (p.T4150A) which is a VUS	Based on information from ClinVar, pubmed, and data from our cohort, c.12448A>G (p.T4150A) is a likely pathogenic variant.	Case is considered solved
